# Comparative analysis of adenovirus and rotavirus gastroenteritis: insights into inflammatory response and temporal variations

**DOI:** 10.3389/fped.2025.1523531

**Published:** 2025-03-31

**Authors:** Pınar Önal, Abdülkadir Bozaykut

**Affiliations:** Department of Pediatrics, Zeynep Kamil Maternity and Children's Disease Training and Research Hospital, University of Health Sciences, Istanbul, Türkiye

**Keywords:** children, rotavirus, adenovirus, mean platelet volume, neutrophil/lymphocyte value, gastroenteritis

## Abstract

**Background:**

Acute viral gastroenteritis remains a leading cause of healthcare costs globally, prompting the need for effective yet simple diagnostic strategies. This study aimed to comprehensively examine the demographic, clinical, and seasonal characteristics, hemogram parameters, and C-reactive protein levels in children diagnosed with adenovirus and rotavirus gastroenteritis.

**Methods:**

By conducting a retrospective study, three groups of children diagnosed with gastroenteritis (Rotavirus positive, adenovirus positive, and rota/adenovirus negative group) were compared in terms of demographic, clinical, and laboratory parameters.

**Results:**

Among 265 children with gastroenteritis, 59.6% were under 36 months, and 36.8% of this group tested positive for rotavirus, while 30% of them tested positive for adenovirus. A statistically significant association was observed between rotavirus gastroenteritis and high neutrophil/lymphocyte value (2.76 ± 1.52) and decreased mean platelet volume (7.47 ± 0.36 fL). Temporal analysis revealed numerical peaks in the adenovirus group in summer and rotavirus in winter; however, these differences were not statistically significant (*p* = 0.684, 0.851). Unlike rotavirus, our study did not reveal any prominent laboratory marker that serves as a distinctive feature of adenovirus.

**Conclusions:**

Our findings suggested that decreased mean platelet volume and high neutrophil/lymphocyte ratio were found statistically significant in rotavirus gastroenteritis, distinguishing it from other causes of acute gastroenteritis.

## 1 Introduction

Acute gastroenteritis (AG) is a globally prevalent condition that poses a significant burden on the health and well-being of children worldwide (1). It remains a leading cause of morbidity and can result in severe dehydration, hospitalization, and even mortality, particularly in developing countries where healthcare resources may be limited. According to the World Health Organization, AG is responsible for approximately 1.7 billion cases of diarrhea annually, leading to nearly 525,000 deaths in children under five years old age (2). The etiology of AG in children predominantly includes bacterial, viral, and parasitic infections, with rotavirus, adenovirus, astrovirus, and norovirus being the primary culprits. These pathogens are typically transmitted through the fecal-oral route, facilitated by the ingestion of contaminated food or water, poor hygiene practices, and close contact with infected individuals. Despite significant advances in public health interventions, such as the implementation of rotavirus vaccines, viral gastroenteritis remains a substantial public health challenge, particularly in regions where vaccine coverage is low or sanitation infrastructure is lacking (3–5). The clinical presentation of AG is characterized by symptoms such as vomiting, diarrhea, abdominal pain, and fever. Differentiating viral etiologies from other causes based solely on clinical symptoms is challenging due to the overlap in presentation. Diagnostic confirmation often relies on various laboratory methods, including culture, molecular, and serological techniques (5). However, these diagnostic approaches may be less accessible in resource-limited settings, particularly in rural or remote areas. Basic laboratory tests, such as hemogram and acute phase reactants, can be invaluable to clinicians in such contexts. Acute phase reactants are proteins synthesized by the liver in response to inflammation, trauma, or tissue injury, and they can be categorized into positive and negative reactants. Positive acute phase reactants, including C-reactive protein (CRP) and fibrinogen, increase concentration during inflammatory states. Conversely, negative reactants, such as albumin and transferrin, decrease in response to inflammation (5–7). Elevated CRP levels indicate ongoing inflammation and are widely utilized in diagnosing and monitoring inflammatory conditions. In the context of AG, the immune system mounts a strong response, resulting in the production of markers like CRP. Given the global burden of viral gastroenteritis and the limitations of current diagnostic approaches in resource-limited settings, there is a critical need for studies exploring the utility of readily available biomarkers, such as hemogram parameters and CRP, in diagnosing and managing AG. This study aimed to assess demographic, clinical characteristics, and laboratory evaluations of children diagnosed with acute gastroenteritis.

## 2 Materials and methods

### 2.1 Study design

This retrospective study was conducted at the Zeynep Kamil Maternity and Children's Diseases Training and Research Hospital between January 2016 and December 2016. Ethical Approval for the study was obtained from the institutional ethics committee (Approval Date: 15.09.2017, Approval No: 132). The study included pediatric patients aged 1 month to 18 years who presented to the emergency department with acute gastroenteritis (AG) diagnosis. Subsequently, children under 36 months were selected for a detailed analysis. Inclusion criteria were the presence of diarrhea (defined as loose or watery stools occurring more than three times per day) with a duration of less than two weeks, and complete laboratory evaluation including rotavirus and adenovirus antigen tests, hemogram, and C-reactive protein (CRP) levels. Patients with positive bacterial cultures in stool samples, those with underlying chronic diseases, and those with coinfections were excluded from the study.

Additionally, children with positive results for both adenovirus and rotavirus antigens in stool samples were excluded to avoid confounding factors. Adenovirus and rotavirus antigens were detected in stool samples using the immunochromatographic method (CİTEST, Rotavirus/Adenovirus Rapid Test, England). After the exclusion of patients with positive bacterial cultures, the remaining children with AG were categorized into three groups based on their antigen test results: Adenovirus positive (AP), Rotavirus positive (RP), and Adenovirus/rotavirus negative (ARN). The demographical data and laboratory parameters (hemoglobin, white blood cell, neutrophil, lymphocyte, platelet count, neutrophil/lymphocyte ratio, mean cell volume, and mean platelet volume) were compared. The distribution of dehydration severity among RP, AP, and ARN groups were classified as mild, moderate, and severe (8).

### 2.2 Statistical analysis

Data were analyzed using the Statistical Package for Social Sciences (SPSS, version 26). Numerical data fitting a normal distribution were presented as mean ± standard deviation (SD), while categorical variables were expressed as numbers and percentages. The Kolmogorov–Smirnov test was used to assess the normality of the data distribution. One-way ANOVA and chi-square tests were used to compare the groups. A *p*-value of <0.05 was considered statistically significant. For *post-hoc* analysis, Bonferroni correction was used.

## 3 Results

### 3.1 Demographic and clinical characteristics of children

**Figure 1:**
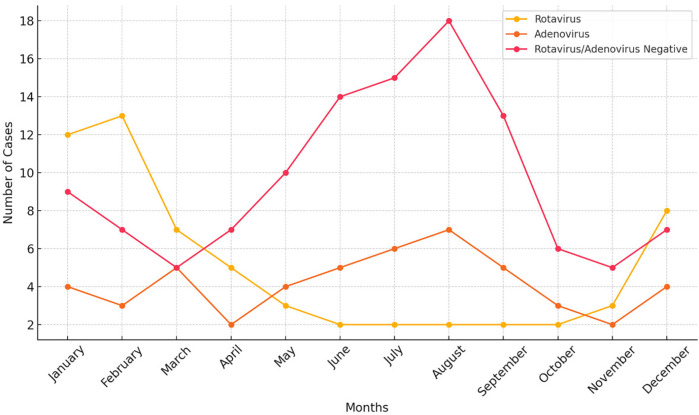
Distribution of three groups by months.

During the study period, a total of 265 children presented to the emergency department with a diagnosis of acute gastroenteritis (AG). Patients with chronic illnesses, a history of trauma, other inflammatory conditions, or those on regular medication or children diagnosed with bacterial or parasitic forms of AG (*n*: 42) were excluded from the analysis. Subsequently, among the remaining 223 patients, those under 36 months were included in the study (133 children, 59.6%). Among these patients, 49 (36.8%) children were positive for rotavirus antigen, while 40 (30.1%) children had a positive stool antigen test for adenovirus. A total of 44 (33.1%) children tested negative for both adenovirus and rotavirus. The study population had a mean age of 15.4 ± 11.39 months and consisted of 67 (50.3.%) female and 66 (49.7%) male patients. The Adenovirus positive and ARN groups showed a peak in summer, whereas the RP group showed a peak in the winter season (Figure 1). The analysis of the monthly distribution of AP, RP, and ARN groups revealed no statistically significant differences, with *p*-values of 0.684, 0.851, and 0.900, respectively.

### 3.2 Comparison of group features

The distribution of dehydration severity among RP, AP, and ARN groups were classified as mild, moderate, and severe. Following results were observed: Rotavirus positive group (mild: 27, moderate: 15, severe: 7), AP group (mild: 19, moderate: 13, severe: 8); and ARN group (mild: 22, moderate: 18, severe: 6). The chi-square test revealed no statistically significant difference in the distribution of dehydration severity among these three groups (*p* = 0.803), indicating that dehydration severity was similarly distributed across the RP, AP, and ARN groups.

Vomiting was observed in 24 out of 49 patients in the RP group, 17 out of 40 patients in the AP group, and 23 out of 44 patients in the ARN group. There was no statistically significant difference among the groups (*p* = 0.662). Fever was present in 17 out of 49 patients in the RP group, 20 out of 40 patients in the AP group, and 15 out of 44 patients in the ARN group. Similarly, there was no statistically significant difference across the groups (*p* = 0.239).

**Figure 2:**
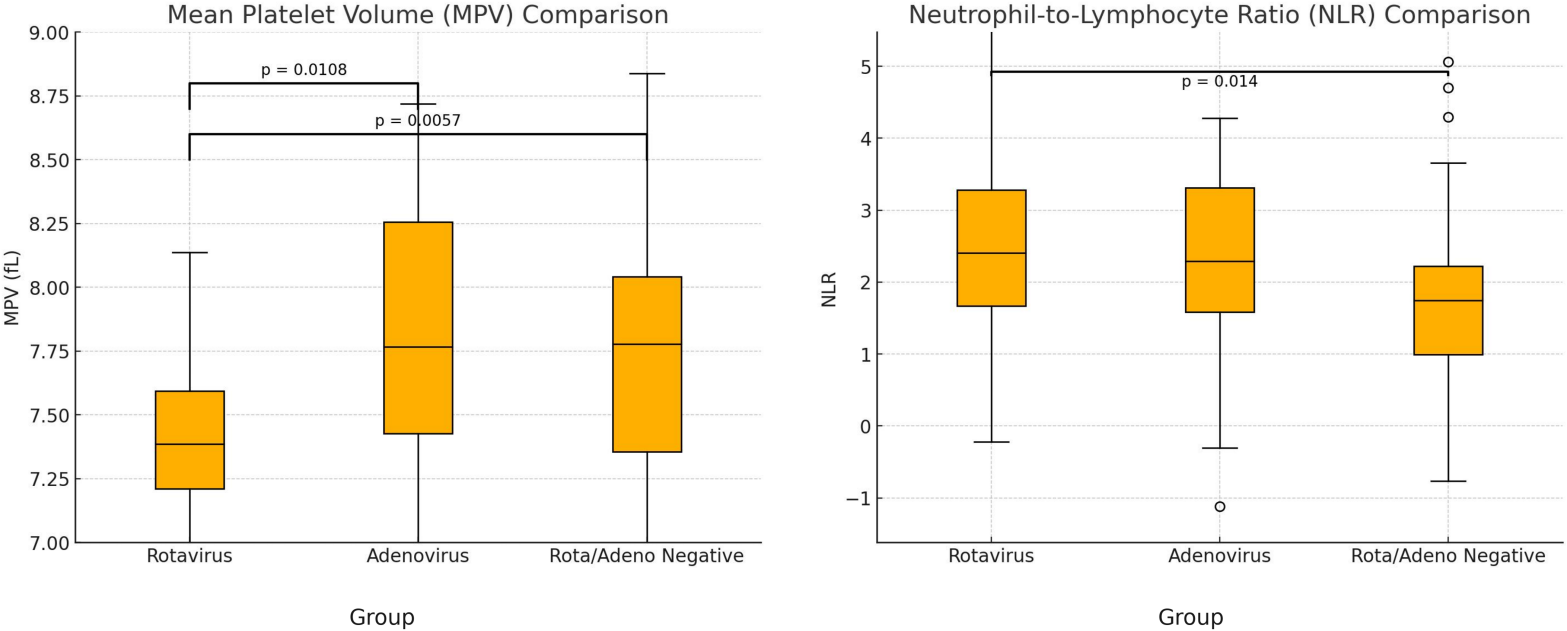
Comparison of mean platelet volume (MPV) and neutrophil/lymphocyte ratio (NLR). (*P*-value with Bonferroni correction was found as 0.0167).

Total rotavirus vaccination rate was 15.7%. A comparative analysis between the groups was summarized in Table 1. The mean platelet volume values of the three groups were compared with one-way ANOVA test, which revealed a statistically significant difference (*p* = 0.0023). *Post-hoc* analysis showed that MPV was significantly lower in the RP group than in the AP group (*p* = 0.0108) and the ARN group (*p* = 0.0057) (Figure 2). However, there was no significant difference between the AP and ARN groups (*p* = 0.99). The Bonferroni correction, used for the evaluation of *post-hoc* pairwise comparisons. The *p*-value threshold was determined to be 0.0167. The mean NLR values were present in Table 1. One-way ANOVA showed a statistically significant difference among the groups (*p* = 0.0250). *Post-hoc* analysis indicated that NLR was significantly higher in the RP group compared to the ARN group (*p* = 0.014). The differences between the RP and AP groups (*p* = 0.045) and the AP and ARN groups (*p* = 0.302) were not statistically significant.

**Table 1:** Comparative assessment of demographic and laboratory features within groups.

Patient group	Rotavirus positive^a^	Adenovirus positive^a^	Rota/adeno negative^a^	*p*
Age (months)	15.77 ± 10.87	15.07 ± 10.84	15.51 ± 12.48	0.25
C-reactive protein (mg/dl)	1.66 ± 1.47	0.80 ± 0.55	0.92 ± 0.63	0.81
Hemoglobin (g/dl)	12.03 ± 1.16	11.77 ± 1.47	11.86 ± 1.10	0.58
Neutrophil(10^9^/L)	5.63 ± 4.3	4.62 ± 4.05	5.65 ± 4.86	0.32
Lymphocyte(10^9^/L)	2.05 ± 0.9	2.7 ± 1.34	3.21 ± 1.79	0.034
Platelet count(10^9^/L)	281.140 ± 74.159	292.631 ± 64.654	343.139 ± 46.522	0.38
Neutrophil/Lymphocyte	2.76 ± 1.52	2.26 ± 1.29	1.79 ± 1.33	0.025
Mean cell volume (fl)	75.35 ± 4.9	76.23 ± 5.8	74.1 ± 6.5	0.51
Mean platelet vol (fl)	7.47 ± 0.36	7.75 ± 0.62	7.8 ± 0.74	0.0023

^a^ mean ± standard deviation.

## 4 Discussion

Acute viral gastroenteritis remains a significant morbidity, healthcare cost, and mortality, especially in developing countries with limited access to clean water and healthcare facilities. Rotavirus was the most common cause of gastroenteritis in children under 36 months (36.8%) in our study. It is a highly contagious, vaccine-preventable virus that affects infants and young children and is a leading cause of severe diarrhea, dehydration, hospitalization, and even mortality worldwide. In some regions where rotavirus vaccination is a part of the national immunization program, a decline in rotavirus incidence has been reported (9). A large-scale global study investigating the burden of rotavirus reported that the incidence of rotavirus infection ranged from 0.024 to 1.63 cases per child-year. The highest incidence was observed in the Democratic Republic of the Congo (1.63 cases per child-year), where rotavirus vaccination programs are not implemented, and sanitation conditions are suboptimal. In our country, the incidence was reported as 0.25 cases per child-year, which is also higher than in high-income countries where routine rotavirus vaccination is implemented (1). Increasing vaccination coverage is extremely important to prevent complications and reduce mortality. In our study, the rotavirus vaccination rate was 15.8%, which is lower than the rate reported in the study by Gönüllü et al. from our country (10).

It is well-known that rotavirus gastroenteritis peaks between December and April, whereas adenovirus gastroenteritis can be observed throughout the year (11, 12). Studies conducted in countries where rotavirus has been incorporated into the national vaccination program have reported a decline in case numbers following vaccination and a shift of the peak season to a later period, delayed by eight to fifteen weeks (13). Our study revealed that rotavirus diarrhea has a peak in the winter season in our study. In contrast, although not statistically significant, the ARN group showed a noticeable peak during the summer season (Figure 1). This observation may be explained by the presence of other viral agents, such as astrovirus or norovirus, which are known to cause outbreaks specifically in the summer season, as similarly reported in a study conducted in Lebanon (14). Rotavirus and adenovirus gastroenteritis predominantly affect children between six months and two years of age. Consistent with this data, over sixty percent of the patient cohort comprised children under the age of three.

Complete blood count and acute phase reactants can provide clinicians insights into rotavirus and adenovirus gastroenteritis. Subsequently, studies investigating laboratory markers in AG focus on hemogram parameters such as mean platelet volume because of their simplicity and low price (15). For example, MPV refers to the average size of platelets in blood and is an important indicator of platelet activity and function. MPV value seems to be influenced by many factors, such as connective tissue diseases, smoking, cerebrovascular diseases, or inflammation (16). Yet, there is a paucity of literature about the infection-MPV relationship. In our study, mean MPV value was statistically significantly low in the RP group. Mete et al. (2014) and Zhang et al. (2020) found that patients with rotavirus gastroenteritis have lower MPV values than control groups in their studies, similar to us (17, 18). In another study, low MPV level was found to be correlated with *Entamoeba histolytica* gastroenteritis compared to control group (19). However, there are some reports on the MPV-infection relationship that have yielded inconsistent results. Such as, MPV was found to increase urinary tract infections and hepatitis B (20, 21). These discrepancies among MPV values may be associated with the degree of inflammation. The alteration in MPV value is thought to result from the thrombopoiesis process. In cases of mild inflammation, it's suggested that MPV increases due to the larger size of newly produced platelets, while in high-grade inflammation, MPV is claimed to decrease due to platelet consumption (16). Lymphopenia and viral infections often have a close association cause some viruses can damage lymphocyte production and function. Many investigations have probed the intricate relationship between viruses and lymphocyte count, yielding divergent mechanisms such as cell death, elevated cytokines, or inhibition of lymphopoiesis (22). Our findings demonstrated lymphopenia was detected more in patients with rotavirus gastroenteritis, than other groups which is consistent with the data presented by Zhang et al., who also observed decreased lymphocyte and MPV values more in rotavirus gastroenteritis than control groups (18). Conversely, Wang et al. (2007) (23) highlighted the lack of comprehensive data on lymphocyte regulation in rotavirus gastroenteritis, pointing to B-cell activation and altered T-lymphocyte function in this group. Hence, further investigations about lymphocyte subtypes in rotavirus infection are warranted to elucidate the short and long-term effects of rotavirus on the immune system. Neutrophil/lymphocyte ratio (NLR)stands as a simple and cost-effective marker that reflects the severity of immune-inflammatory reactions. Recent studies have demonstrated that the NLR was higher in liver disease, rheumatic diseases, and influenza virus compared to control groups (24–26). Moreover, certain studies have identified a relationship between the level of NLR and the intensity of inflammatory response. It has been stated the NLR is around 2–3 in low-grade inflammation, whereas it's above 3 in higher-grade inflammation (27). A high NLR value (2.8 ± 2.1) was remarkable in the RP group in our research. In line with our results, Çelik et al. reported a higher NLR in rotavirus gastroenteritis rather than adenovirus (28). Meanwhile, compared to our results, Çelik et al. reported a higher mean NLR (4.09 ± 5.3). These variations might be associated with the diverse spectrum of severity of gastroenteritis.

Focusing on the adenovirus positive group in our research, we should point out that no statistically significant data was found regarding demographic or clinical data except for seasonality distinctions. Adenovirus is a type of DNA virus capable of causing various 220 diseases, including respiratory tract infections, conjunctivitis, and gastroenteritis following rotavirus. Nascimento et al. (2017) reported that adenovirus gastroenteritis occurs most frequently in children aged 6–24 months, with statistical significance (29). In our research, although adenovirus was most commonly seen between 0 and 6 months of age, no statistically significant difference was found comparing other age groups. Additionally, unlike rotavirus, our study did not reveal any prominent laboratory marker that serves as a distinctive feature of adenovirus. In fact, there was little knowledge about the MPV-adenovirus relationship or hematological parameters in adenovirus AG in the literature.

While our study provided some valuable insights, it's important to acknowledge limitations such as retrospective nature. Also, the adenovirus and rotavirus-negative groups likely consisted of other viruses, such as norovirus and astrovirus, which we were unable to investigate. Despite these limitations, our study contributed to the growing body of literature by demonstrating that MPV values are significantly low while NLR is high in cases of rotavirus gastroenteritis. For future studies, findings from different markers in blood or stool and serial MPV measurements would contribute to a more comprehensive understanding of viral gastroenteritis.

## Data Availability

The datasets presented in this article are not readily available because data is restricted due to patients' confidentiality. Requests to access the datasets should be directed to drpinaronal@gmail.com.
